# Obituary

**Published:** 1880-09

**Authors:** Robert Arthur


### OBITUARY.

### ROBERT ARTHUR, M.D., D.D.S

ROBERT ARTHUR, son of William and Anna Arthur, was born at Calverton, near Baltimore, Md., July 22, 1819, and died June 22, 1880, at Baltimore, of embolism, which occluded the main arteries of his right leg, resulting in gangrene. His early education was received at ordinary private schools near his home. At the age of about fifteen he was thrown upon his own resources, and entered a printing-office. He was at length induced to commence the study of dentistry, when he placed himself under the instruction of Dr. Chapin A. Harris, and entered the Baltimore College of Dental Surgery. In due time he graduated, receiving the first degree of D.D.S. ever conferred. During the period between leaving school and his graduation in 1841, he by study and a wide course of reading acquired a good education, which extended to the languages,—Latin, Greek, French, and German. He practiced for a short time after this latter period in Philadelphia, then removed to Baltimore, practicing in winter and spending his summers itinerating among the leading planters of Virginia. On his marriage, in 1847, to Mary Hemple, of Philadelphia, he opened an office in Washington, D. C., where he practiced with eminent success for seven years. At this time the honorary degree of M.D. was conferred upon him by the Washington University of Baltimore. He was largely instrumental in the organization of the Philadelphia College of

Dental Surgery, which was established in 1852, and of which he filled the chair of Principles of Dental Surgery. He gave up his practice in Washington in 1854, and removed to Philadelphia to be near the school.

His most distinguishing moral characteristics were his strict integrity and conscientiousness, as it was impossible for him to admit, or even tacitly acquiesce in, any act that was tainted with the least suspicion of dishonor. He was naturally of gentle demeanor and of retiring disposition, but had the boldness and courage of the lion when aroused by any wrong, or when the public good seemed to him to require defense. His most marked mental qualities were the energy with which he held his views, and the full, clear, and concise terms in which he expressed them. His physical health was generally good, but his nervous tone frequently was reduced to zero by the intensity of his mental action, combined with the exhaustive nature of his daily work; but for this his literary work had been greatly larger and even more conspicuous. It must be conceded that wherever the influence of dental literature has extended, the moral force and views of Dr. Arthur have commanded consideration and respect, and that his suggestions have modified the practice of dentistry to no inconsiderable degree.—*Dental Cosmos.*

